# A Biodegradable Polyester-Based Polymer Electrolyte for Solid-State Lithium Batteries

**DOI:** 10.3390/nano13233027

**Published:** 2023-11-27

**Authors:** Chenxia Tang, Zhiyu Xue, Shijie Weng, Wenjie Wang, Hongmei Shen, Yong Xiang, Le Liu, Xiaoli Peng

**Affiliations:** 1 School of Materials and Energy, University of Electronic Science and Technology of China, Chengdu 611731, China; 202211030527@std.uestc.edu.cn (C.T.); 202221030328@std.uestc.edu.cn (S.W.); 202121030330@std.uestc.edu.cn (W.W.); 202121030329@std.uestc.edu.cn (H.S.); xiang@uestc.edu.cn (Y.X.); 2 Advanced Energy Research Institute, University of Electronic Science and Technology of China, Chengdu 611731, China; 3 Sichuan Provincial Engineering Research Center of Flexible Display Material Genome, University of Electronic Science and Technology of China, Chengdu 611731, China; 4 School of Mechanical and Electrical Engineering, Wuhan Institute of Technology, Wuhan 430200, China; zq@uestc.edu.cn

**Keywords:** lithium metal batteries, solid polymer electrolyte, polyester polymers, biodegradable

## Abstract

The low ionic conductivity, narrow electrochemical window, poor interfacial stability with lithium metal, and non-degradability of raw materials are the main problems of solid polymer electrolytes, restricting the development of lithium solid-state batteries. In this paper, a biodegradable poly (2,3-butanediol/1,3-propanediol/succinic acid/sebacic acid/itaconic acid) ester was designed and used as a substrate to prepare biodegradable polyester solid polymer electrolytes for solid-state lithium batteries using a simple solution-casting method. A large number of ester-based polar groups in the amorphous polymer become a high-speed channel for carrying lithium ions as a weak coordination site. The biodegradable polyester solid polymer electrolyte exhibits a wide electrochemical window of 5.08 V (vs. Li/Li^+^), high ionic conductivity of 1.03 mS cm^−1^ (25 °C), and a large Li^+^ transference number of 0.56. The electrolyte exhibits good interfacial stability with lithium, with stable Li plating/stripping behavior at room temperature over 2100 h. This design strategy for biodegradable polyester solid polymer electrolytes offers new possibilities for the development of matrix materials for environmentally friendly lithium metal solid-state batteries.

## 1. Introduction

Liquid organic electrolytes used in commercial lithium rechargeable batteries are severely limited by their high flammability, potential leakage, and non-degradability [1,2,3,4]. The discovery of solid electrolytes offers new ideas for improving the safety of batteries because they are less flammable, do not leak, and are safer than liquid electrolytes. All-solid electrolytes can be classified into solid polymer electrolytes (SPEs) and inorganic solid electrolytes (ISEs) [5,6]. Despite the advantages of ISEs, complicated preparation processes and large interfacial independence with the electrode severely limit their development [7,8]. Compared with ISEs, SPEs exhibit superior flexibility, lower interface resistance, and intimate contact with electrodes. Although SPEs have made significant progress in recent years, they still have problems in ionic conductivity, electrochemical window and sustainability, and environmental compatibility.

Polyethylene oxide (PEO) is the most widely studied structure because the ether bonds inside PEO can form a stable complex with lithium ions for their transport [9,10,11,12]. However, PEO-based solid electrolytes exhibit low ionic conductivity at room temperature (mainly related to the high crystallinity of the polymer matrix) and have a narrow electrochemical window (<3.9 V), restricting their practical application [13,14,15,16,17]. Polyester polymer is a polymer electrolyte matrix newly developed in recent years. Like the ether–oxygen bond in PEO, the ester group contained in the polyester polymer matrix can serve as a coordination site for lithium ions. Strong coordination occurs between the ether–oxygen bond and the lithium-ion PEO-based solid electrolyte; thus, the migration speed of lithium ions is slowed down. Meanwhile, the complexation between the ester or the ketone group and lithium ions is weak, and each coordination point of the ester group carries a lithium ion. Fast ion channeling is conducive to the migration of lithium ions [4,18,19,20,21]. However, the low concentration of the polar group C=O cannot effectively solvate the cation, the T_g_ value and crystallinity are usually high, the movement of the polymer segment is restricted, and the LUMO and HOMO energy levels do not satisfy the interfacial stability of the anode/cathode, resulting in the low ionic conductivity and narrow electrochemical window of polyester-based solid electrolytes [22,23,24]. Millions of tons of lithium batteries will be retired in the next few years, and most polymer matrices are usually difficult to degrade, which poses challenges for the recycling of lithium batteries; thus, they have a negative impact on the environment. 

Many efforts have been made to overcome this predicament. Shriver and Wei [25] used the strategy of increasing the number of ester groups and disrupting the polymer order and successfully synthesized polyethylene carbonate (PVIC) and its derivatives, poly (1,3-dioxolan-2-one-4,5-diyl oxalate) (PVICOX), for the first time. Ionic conductivity can reach 0.1 mS cm^−1^ at ambient temperature. Zhang and coworkers [26] reported a poly (allyl acetoacetate) (PAAA)-based polymer electrolyte using UV curing technology; ionic conductivity at 30 °C reached 0.11 mS cm^−1^ and the electrochemical window reached 4.8 V. Thus, good interface compatibility with LiFePO_4_ and Li_1.2_Ni_0.2_Mn_0.6_O_2_ high-voltage cathode is exhibited. However, due to the reverse migration of the TFSI anion at room temperature, the ion migration number of the PAAA polymer electrolyte can only reach 0.37. Cui et al. [7] prepared a PVCA (poly (vinylene carbonate)) polymer electrolyte using in situ synthesis, which yielded a high electrochemical window (4.5 V) and low ionic activation energy (0.03 eV). However, the excessive regularity of the PVCA chain segment limited its mobility, resulting in low ionic conductivity (0.0982 mS cm^−1^ at 50 °C). Mindemark et al. [27] synthesized poly (trimethylene carbonate) (PTMC), and the T_g_ value and ionic conductivity were optimized by adjusting the ratio of trimethylene carbonate (TMC) and e-caprolactone (CL); it was found to have a T_g_ of −26 °C and a conductivity of 0.016 mS cm^−1^ at 60 °C. To further improve ionic conductivity, electrochemical window, ion transference number, efficient recovery, and utilization performance of polyester-based solid electrolytes, it is necessary to optimize the polymer segment structure; inhibit crystallization; and use renewable, degradable, and low-toxicity monomer sources. 

In this context, a biodegradable poly (2,3-butanediol/1,3-propanediol/succinic acid/sebacic acid/itaconic acid) ester (BPE) was designed for application in polymer electrolyte matrices. The design of the BPE is based on the following considerations: (1) In order to meet the characteristics of low toxicity, renewability, and degradability, all monomers (2,3-butanediol (2,3-BDO)/1,3-propanediol (1,3-PDO)/succinic acid (SuA)/sebacic acid (SeA)/itaconic acid (IA)) are derived from renewable biomass available for mass production [28]. Therefore, the BPE exhibits good biodegradability. (2) SuA and IA with short backbones are used in order to introduce more donors of ester bonding (-C=O-) groups that can form coordinate bonds with Li cations for fast ion transport [29,30]. (3) Random copolymerization and introducing a methyl side group in the backbone are used as strategies for breaking the regularity of the polymer, increasing the content of the amorphous region, which is conducive to the rapid transport of lithium ions. (4) The introduction of long carbon chains is beneficial to the stability of lithium and improves the electrochemical stability of lithium batteries. Bio-based polymer solid-state electrolytes (BPSPEs) are prepared by mixing the BPE with LiTFSI using a simple solution-casting method. As the results confirm, BPSPEs exhibit high ionic conductivity of 1.03 mS cm^−1^ (25 °C), a large Li^+^ transference number of 0.56, and a wide electrochemical window of 5.08 V (vs. Li/Li^+^). The electrolyte demonstrates good interfacial stability with lithium, exhibiting stable Li plating/stripping behavior at room temperature over 2100 h. At a current rate of 0.1 C, the solid-state Li (Ni_0.8_Co_0.1_Mn_0.1_) O_2_/Li battery exhibits a high initial capacity of 191.7 mAh g^−1^ at room temperature. After 100 cycles, the capacity exceeds 128.2 mAh g^−1^. Polyester solid electrolytes provide good thermodynamic stability and cycling performance, have simple preparation, and do not exert adverse effects such as pollution or degradation. The design strategy of BPE provides additional options for the development of new bio-based degradable functional materials such as lithium-ion conductors.

## 2. Materials and Methods

### 2.1. Synthesis of Biodegradable Poly (2,3-Butanediol/1,3-Propanediol/Succinic Acid/Sebacic Acid/Itaconic Acid) Ester (BPE) 

**Figure 1:**
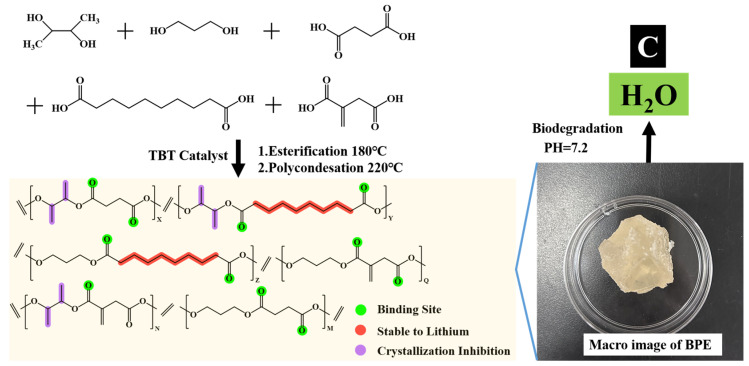
The synthetic route of BPE.

The synthetic procedure for the preparation of BPE is illustrated in Figure 1. The following reagents were used: 2,3-butanediol (2,3-BDO), 1,3-propanediol (1,3-PDO), selenoacetamide (SeA), sulfonamide (SuA), imidazole acetate (IA), phosphite. These reagents were obtained from commercial sources, specifically Alfa Aesar (Heysham, UK), Beijing Chemical Reagent Company (Beijing, China), and Tianjin Light Recovery Reagent Institute (Tianjin, China). Reactants 2,3-BDO (0.25 mol, 22.50 g), 1,3-PDO (0.25 mol, 19.00 g), SeA (0.225 mol, 45.45 g), SuA (0.225 mol, 26.55 g), IA (0.05 mol, 6.50 g) were mixed in a 100 mL three-necked flask equipped with a mechanical stirring device, a recirculating condensing unit and a thermometer. Following four cycles of nitrogen replacement, the reaction mixture was subjected to heating in an oil bath under a nitrogen atmosphere at 180 °C for a duration of 2 h. Upon the cooling of the reaction mixture to below 100 °C, the catalyst butyl titanate (obtained from Alfa Aesar, Heysham, UK) was introduced. Subsequently, the reaction mixture was gradually heated to 220 °C while the pressure was simultaneously reduced to a vacuum. The reaction was then sustained for a duration of 4−6 h under these conditions. Upon the observation of the “Weissenberg” effect in the reaction mixture, the stirring rate and temperature were gradually decreased. The product was then collected after the reaction temperature cooled to below 60 °C. Subsequently, the reaction product was dissolved in trichloromethane (obtained from Beijing Chemical Plant, Beijing, China) to eliminate any unreacted monomer and oligomer. The solution was then precipitated using an excess of cold methanol (analytically pure, from Beijing Chemical Plant). Finally, the obtained precipitate was subjected to drying at 60 °C until constant weight was attained.

### 2.2. Assembly of Solid-State Batteries

#### 2.2.1. Preparation of BPSPE

After the BPE was dissolved in N-methyl-2-pyrrolidone (NMP) and mixed with LiTFSI, the proportions of lithium salt to the total mass of the polymer electrolyte were 16.7, 44.4, 58.3, and 61.5 wt.%. The slurry was scraped onto a positive shell of a coin cell, and dried at 45 °C for 2 h and 80 °C for 24 h to obtain BPSPEx film (x = 16.7, 44.4, 58.3 and 61.5) for EIS, LSV testing. The slurry was scraped onto a separator with a thickness of 7 microns, and dried at 45 °C for 2 h and 80 °C for 24 h to obtain a separator-supported BPSPE_44.4_ for NCM811/BPSPE_44.4_/Li solid coin cell and a pouch-type stacking full cell.

#### 2.2.2. Coin Cell

For the NCM811/BPSPE_44.4_/Li solid battery, the active material ratio of the NCM811 (3–4.3 V) (Guangdong Canrd New Energy Technology Co., Ltd., Dongguan, China) conventional cathode electrode was 94.5%. The active material mass loading of the conventional NCM811 electrode was relatively high at 19.8 mg cm^−2^. Because there are no solid electrolytes in a conventional NCM811 electrode, a 80 μL liquid electrolyte LB111 (Suzhou DoDoChem Technology Co., Ltd., Suzhou, China) was dropped onto the conventional NCM811 electrode to enhance the ion transport of lithium ions inside the high-mass loading cathode. CR 2032 coin-type cells were assembled in an Ar-filled glovebox with Li metal foils as the anode, separator-supported BPSPE_44.4_, and a high-mass loading conventional NCM811 electrode as cathode.

#### 2.2.3. Pouch-Type Stacking Full Cell

The LiCoO_2_ (4.5 V) cathode was composed of an 80 wt.% LiCoO_2_, a 10 wt.% Super P, and a 10 wt.% binder (BPSPE_44.4_). The mass loading of active materials was approximately 15 mg cm^−2^. The graphite anode was composed of an 80 wt.% graphite, a 10 wt.% SP, a 10 wt.% binder (BPSPE_44.4_). After stacking the LiCoO_2_ (4.5 V) cathode, the graphite anode, and the separator-supported BPSPE_44.4_, the positive and negative electrode ears were welded, and the cell was sealed with aluminum plastic film. 

### 2.3. Materials Characterization

The thermogravimetric analysis curve of BPE was obtained by the con increasing the temperature from 25 °C to 800 °C at a rate of 10 °C /min in a nitrogen atmosphere. The degradation performance of BPE was tested by placing the sample in a phosphoric acid buffer solution at 37 °C, taking the sample out every five days and weighing it to constant weight in a vacuum oven at 40 °C. Sample degradation performance was calculated using Equation (1):

(1) $$\mathrm{Weight}\;\mathrm{loss}\;(\%)=\frac{W0-W1}{W0}\times 100\%.$$

The test conditions of XRD diffraction were in the angle range of 5–50° at a temperature of 25 °C. Rigaku Ultima IV (Tokyo, Japan). The infrared spectra of BPSPEx (x = 16.7, 44.4, 58.3 and 61.5) solid electrolyte were obtained using an infrared spectrometer (Merryfield IN10, Thermo Fisher Scientific, Waltham, USA). The differential scanning calorimetry (DSC) curve was recorded using a differential scanning calorimetric analyzer (DSC 200F3, Netzsch, Selb, Bavaria, Germany) within a temperature range of −70 °C–70 °C and a heating rate of 10 °C/min. The nuclear magnetic resonance (NMR) carbon spectrum was measured by an NMR spectrometer (JNM-ECZ, JEOL Co., Ltd., Tokyo, Japan), and the solvent used to dissolve the sample was CDCl_3_. 

### 2.4. Electrochemical Characterization

The ionic conductivity of the solid electrolyte from room temperature to 80 °C was characterized by electrochemical impedance spectroscopy (EIS) in stainless steel (SS) /BPSPEx (x = 16.7, 44.4, 58.3 and 61.5)/SS blocking battery in an electrochemical workstation (VersaSTAT3F, Princeton Applied Research, Oak Ridge, TN, USA) within a frequency range of 0.01–10^7^ Hz and an AC amplitude of 50 mV. The ionic conductivity was calculated according to Equation (2), where d is the thickness of the solid electrolyte membrane, *R* is the impedance resistance, and *S* is the effective area of the BPSPE membrane.

(2) $$\Sigma =\frac{d}{RS}.$$

The lithium-ion transference number (t_Li+_) value was determined by a combination of EIS and chronoamperometry (CA) analyses in a Li/BPSPE_44.4_/Li symmetric cell in an electrochemical workstation (PARSTAT 4000 A, Princeton Applied Research, Oak Ridge, TN, USA) and calculated according to Equation (3), where Δ*V* is the applied voltage during CA testing (50 mV), *I*_0_ and *I_s_* are the initial and steady-state current values, respectively, *R*_0_ is the bulk resistance before polarization, and *R_s_* is the steady-state bulk resistance after polarization, as determined by EIS within the frequency range of from 0.01 to 10^5^ Hz.

(3) $$t_{\mathrm{Li}+}=\frac{I_{S}(\Delta V-I_{0}R_{0})}{I_{0}(\Delta V-I_{S}R_{S})}.$$

The electrochemical window of the solid electrolyte was measured by linear sweep voltammetry (LSV) at a sweep rate of 5 mV/s from 0 to 6 V (VersaSTAT3F, Princeton Applied Research) in the SS/BPSPE_44.4_/Li unsymmetrical cell. Li/BPSPE_44.4_/Li and Li/BPSPE_44.4_/NCM811 charge and discharge tests were measured in an automatic galvanostatic charge–discharge unit (Land CT 2001A battery test system, Wuhan Land Electronics Co., Ltd., Wuhan, China).

### 2.5. Analog Computation Details 

A single repeat unit molecular model of the BPE material was constructed to explore the electrochemical properties of solid electrolyte components. These unit components were consistent with the experimentally synthesized raw materials. To mitigate the influence of chain length on electrochemical properties, periodic boundary conditions were applied to x, y, z directions to simulate the infinitely extended spaces [31]. All of density functional theory (DFT) calculations were carried out using the QUICKSTEP program of CP2K [32], which solves the Kohn–Sham equations of DFT by combining a Gaussian basis set for the wave functions with an auxiliary plane wave basis set. We used the 6-31G* basis set together with the B3LYP functional. The energy cut-off for the plane wave basis set was 400 Ry. Based on these optimized geometries, the HOMO–LUMO gaps and electrostatic potential were calculated using the same 6-31G* basis set and B3LYP level. The snapshot and cube files were obtained using the Multiwfn program [33]. Molecular configurations were visualized using Visual MD 1.9.1 [34].

## 3. Results and Discussion

**Figure 2:**
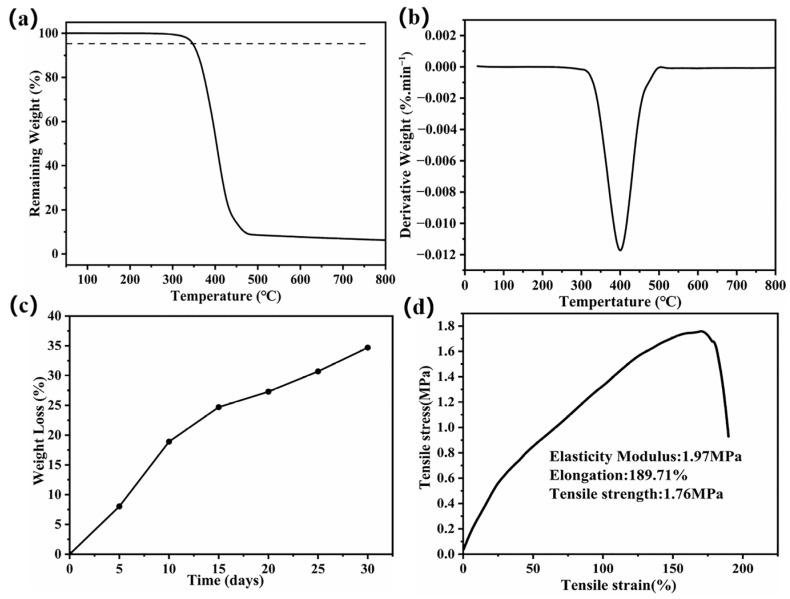
(**a**) Thermogravimetric analysis curve of BPE, (**b**) dynamic thermogravimetric curve of BPE, (**c**) degradation weight loss curves of BPE, (**d**) mechanical tensile curve of BPE.

The thermal stability of BPE is characterized by TGA tests under a nitrogen atmosphere. As shown in Figure 2a, BPE exhibits a one-step decomposition process, i.e., charring of the main chain occurs at 300 °C–600 °C, while the final residue is less than 10% after 800 °C. The decomposition temperature of BPE is 340 °C (Figure 2b), which can meet the thermal stability requirements of most solid electrolytes [35]. Biodegradation is defined as degradation caused by biological activity, particularly significant changes in the chemical structure of a material due to the action of enzymes. The gradual degradation of materials by microorganisms or certain organisms as a source of nutrients leads to loss of quality, loss of properties such as physical properties and ultimately to the breakdown of materials into simpler compounds or monomers. The in vitro degradation property of BPE is depicted in Figure 2c, illustrating a phase of rapid degradation within the initial ten days, primarily characterized by the solubilization of the material and the removal of low-molecular-weight substances. The higher molecular weight segment of BPE exhibits relative stability. Subsequently, the next stage of BPE degradation is relatively gradual. In a degradation process, the ester group of BPE begins to deteriorate, contributing to further degradation of the material [36]. Utilization of degradable materials in lithium batteries holds great significance for battery recycling and environmental protection. The stress–strain curve of the tensile test of BPE film is shown in Figure 2d. The stress and strain exhibit linearity during the initial phase, representing the elastic deformation stage of BPE. BPE exhibits a tensile strength of 1.76 MPa and an elongation at break of 170.26%, meeting the performance requirements of a solid electrolyte matrix. 

**Figure 3:**
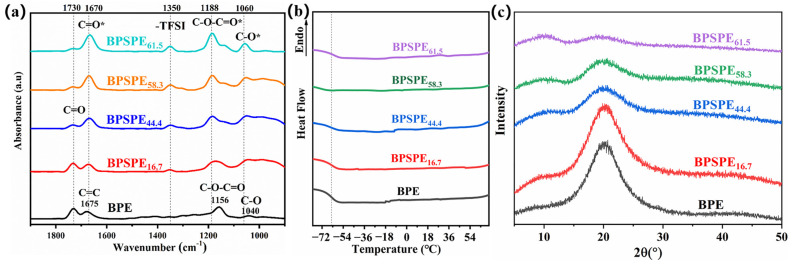
(**a**) Infrared spectra of BPE and BPSPEx (x = 16.7, 44.4, 58.3, 61.5), (**b**) differential scanning calorimetry curves of BPE and BPSPEx (x = 16.7, 44.4, 58.3, 61.5), (**c**) X-ray diffraction curves of BPE and BPSPEx (x = 16.7, 44.4, 58.3, 61.5).

Ion transfer in polymers encompasses a series of processes, including the dissociation of solvated salts, coordination of cations to polar groups, and interchain transfer of ions. To investigate the conduction mechanism of lithium ions in BPE, BPSPEx (x = 16.7, 44.4, 58.3, 61.5) were synthesized using a straightforward solvent pouring method. Comparative infrared spectra of BPSPEx (x = 16.7, 44.4, 58.3, 61.5) with varying LiTFSI concentrations elucidate the interaction process of lithium salts in BPE [37]. The functional groups of BPE and BPSPEx (x = 16.7, 44.4, 58.3, 61.5) are shown in Figure 3a, the “*” symbol denotes the group that interacts with lithium ion and experiences chemical shifts. The addition of lithium salt minimally influences the band position of the IR characteristic bands of BPE. With the gradual increase in lithium salt content, the intensity of the characteristic band of the ester carbonyl group at 1730 cm^−1^ decreases, suggesting a partial reaction between the ester and the lithium salt. This reaction leads to a shifting in the band position [38]. It was observed that the band intensity of the ester carbonyls in BPSPE_58.3_ and BPSPE_61.5_ remained relatively constant. This finding suggests that the interaction between lithium ions and the ester carbonyl group reached a state of saturation, whereby further increases in lithium-ion concentration would not significantly enhance the complexation. It was observed that a portion of the ester carbonyl group at 1170 cm^−1^ was still not redshifted due to the spatial site barrier effect of the double bond, making it challenging for the lithium salt to interact with the ester carbonyl group at this location.

Figure 3b presents the differential scanning calorimetry curves of BPE and BPSPE_X_ (x = 16.7, 44.4, 58.3, 61.5) within the temperature range of −70 °C to 70 °C. It is noted that BPE and BPSPE_X_ (x = 16.7, 44.4, 58.3, 61.5) exhibit amorphous features, indicating that the random copolymerization effectively inhibits the crystallization of BPE. The low glass transition temperature (−60.2 °C) is highly favorable for the transport kinetics of lithium ions in the polymer. Figure 3c shows the XRD spectra of BPE and BPSPEx (x = 16.7, 44.4, 58.3, 61.5), which do not show sharp diffraction peaks, indicating that both BPE and BPSPEx (x = 16.7, 44.4, 58.3, 61.5) exhibit amorphous structures, consistent with DSC results.

**Figure 4:**
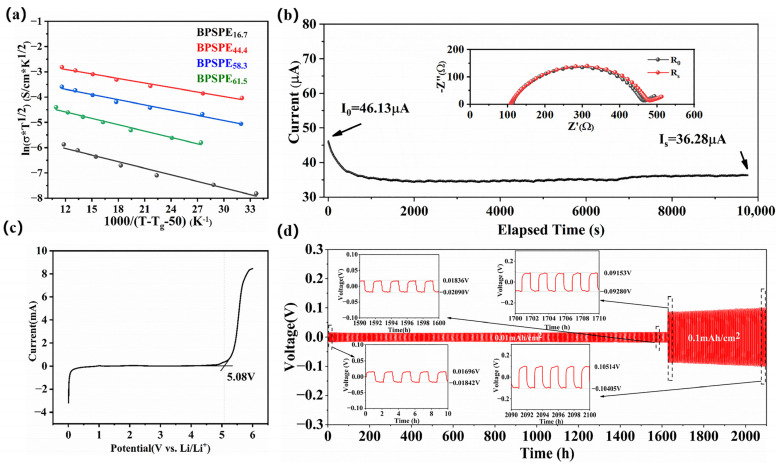
(**a**) VTF curve of BPSPEx (x = 16.7, 44.4, 58.3, 61.5), (**b**) the polarization curve of Li/BPSPE_44.4_/Li symmetrical batteries at room temperature and the energy–quits curve before and after polarization, (**c**) electrochemical windows of BPSPE_44.4_ electrolyte, (**d**) Li/BPSPE_44.4_/Li cells at room temperature at the current density of 0.01 mA cm^−2^ and 0.01 mAh cm^−2^, 0.1 mA cm^−2^ and 0.1 mAh cm^−2^.

Ionic conductivity and ion mobility number are key criteria for evaluating the performance of solid-state electrolytes [39]. The temperature dependent ionic conductivity behavior of BPSPE can be described by fitting it to the Vogel–Tammann–Fulcher (VTF) curve in (Figure 4a), which is described by Equation (4):

(4) $$\sigma (T)={AT}^{1/2}\exp\;(\frac{{-E}_{a}}{R(T-T_{0})}),$$

where *A* is the pre-exponential factor, *E_a_* is activation energy, *T* is the absolute temperature, *T*_0_ is the glass transition temperature in thermodynamic equilibrium statues (*T*_0_ = *T*_g_ − 50), and *R* is the ideal gas constant [40]. The activation energy of BPSPEx (x = 16.7, 44.4, 58.3, 61.5) was determined by fitting the VTF curve (as shown in Table S1). BPSPE_44.4_ showed the lowest activation energy of 0.496 KJ/mol, and the low ion activation makes the movement of the segments easier. The low ionic conductivity of BPSPE_16.9_ is attributed to the low content of lithium ions in BPE. With the increase in lithium salt, BPSPE_44.4_ exhibited a high ionic conductivity, reaching a point where the dissociation of lithium salt was at its maximum. As the lithium salt continued to increase, a polymer electrolyte of the polymer-in-salt system was formed, leading to changes in electrolyte properties. The inhibition of lithium salt dissociation resulted in a decrease in ionic conductivity [41]. The impedance spectra of the blocking electrode/BPSPE_44.4_/blocking electrode system at different temperatures, as illustrated in Figure S1, displayed linear behavior, suggesting the semi-infinite diffusion of lithium ions in BPSPE_44.4_. The intersection of the impedance curve with the Z′ axis represents the total impedance value of the electrolyte sample. The ionic conductivity of BPSPE_44.4_ reached 1.03 mS cm^−1^ at 25 °C. 

The number of ion migrations can characterize the contribution of the charge carriers in the electrolyte to the overall migration of the battery charge. The lithium-ion transference number (t_Li+_) value was determined using Equation (3), and the t_Li+_ of BPSPE_44.4_ was as high as 0.56, effectively reducing the concentration polarization during charge and discharge.

To access the electrochemical stability of the material, Li/BPSPE_44.4_/SS cells were assembled, and the linear sweep voltammograms (LSV) are shown in Figure 4c. No anodic reaction current peaks were observed up to 5.08 V (vs Li/Li^+^), indicating that BPSPE_44.4_ exhibits excellent electrochemical stability and is compatible with most high-voltage cathode materials. In order to analyze the electroplating stripping behavior of lithium metal [42,43] in BPSPE_44.4,_ we conducted an experimental investigation using Li/BPSPE_44.4_/Li batteries, as illustrated in Figure 4d. The experimental protocol involved subjecting the batteries to cycling conditions of 0.01 mA·cm^−2^ and 0.01 mAh·cm^−2^ for 1626 h, followed by 0.1 mA·cm^−2^ and 0.1 mAh·cm^−2^ for additional 476 h at room temperature. This result indicates that the symmetrical cell based on BPSPE_44.4_ displays exceptional overpotential cycling performance, as evidenced by the absence of significant fluctuations after more than 1600 h of operation, and a smaller and flatter voltage hysteresis of approximately 18 mV (at 0.01 mA·cm^−2^ and 0.01 mAh·cm^−2^). Moreover, the cell exhibited stable voltage hysteresis of 105 mV over a period of 476 h (at 0.1 mA·cm^−2^ and 0.1 mAh·cm^−2^). 

**Figure 5:**
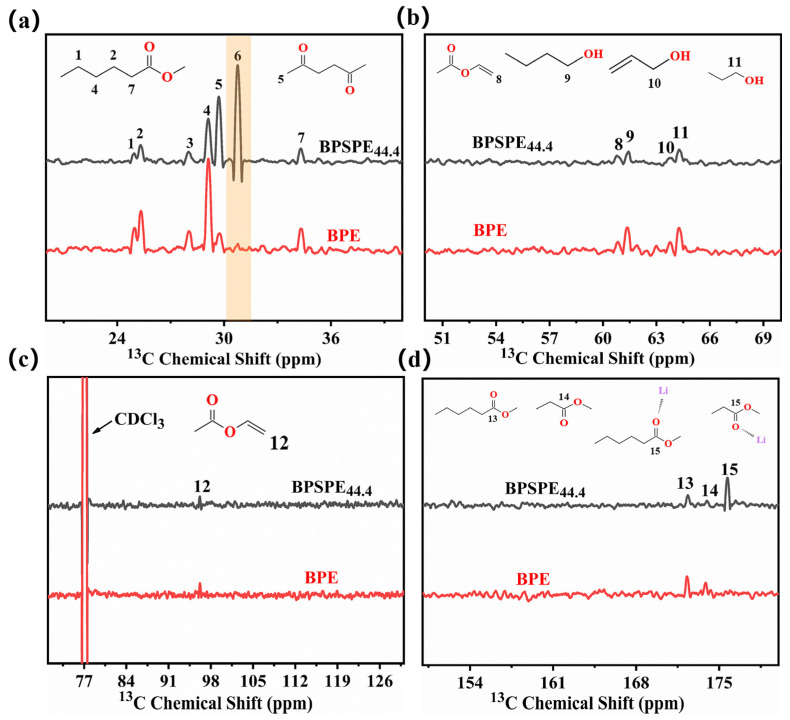
^13^C NMR Spectroscopy of BPE and BPSPE_44.4_ in different chemical displacement intervals: (**a**) 20–40 ppm, (**b**) 50–70 ppm, (**c**) 70–130 ppm, (**d**) 150–180 ppm EIS curves of SS/BPSPE_44.4_/SS batteries at different temperatures.

To investigate the migration mechanism of lithium ions in BPSPE_44.4_, we employed ^13^C NMR carbon spectrum for characterization. Figure 5 shows ^13^C NMR carbon spectra of BPE and BPSPE_44.4_, respectively. According to the chemical displacement, it can be divided into the following four regions: the alkyl region around the ester group (20–40 ppm), the alkyl region around the end chain hydroxyl group (50–70 ppm), the olefin group region around the ester group (70–130 ppm), and the ester group region (150–180 ppm) [44]. As shown in Figure 5a, both samples exhibited identical peak positions at Positions 1 (24.8 ppm), 2 (25.3 ppm), 3 (27.9 ppm), 4 (29.1 ppm), 5 (29.6 ppm) and 7 (34.3 ppm), corresponding to the chemical structure of BPE. The appearance of Peak 6 and the increase in the area of Peak 5 indicated that the complexation of ester groups with LiTFSI caused a chemical shift of adjacent carbon sites to the right. In Figure 5b, Peaks 8–11 corresponded to the structure of the BPE end group. After the addition of LiTFSI, there were no significant changes in Peak 8 and Peaks 9–11; they showed slight area fluctuations. This reflects that the hydroxyl group at the end interacts with TFSI^−^, which is beneficial for the dissociation of LiTFSI and facilitates the migration of lithium ions. The appearance of Peak 12 in Figure 5c indicates that we successfully introduced double bonds: the two samples have less difference in area at this peak, and it is inferred that due to the steric hindrance effect, lithium ions bind less to the ester group at this place. The dynamic process of binding ester-based carbon atoms to lithium ions is shown in Figure 5d. The addition of LiTFSI significantly weakened the characteristic peak of the ester carbon atom, and a new characteristic peak appeared in 175.7 ppm. This indicates that the complexation of the ester group and lithium ions altered the surrounding environment of the ester carbon atoms and increased its chemical displacement. The changes in the chemical environment surrounding the carbon elements in the BPE after the addition of LiTFSI were quantitatively analyzed in Table S2. It is worth noting that while the area of different peaks varies significantly before and after mixing with lithium salts, the total area of peaks remains constant. This indicates that the addition of lithium salts only alters the chemical displacement without masking the signal. 

**Figure 6:**
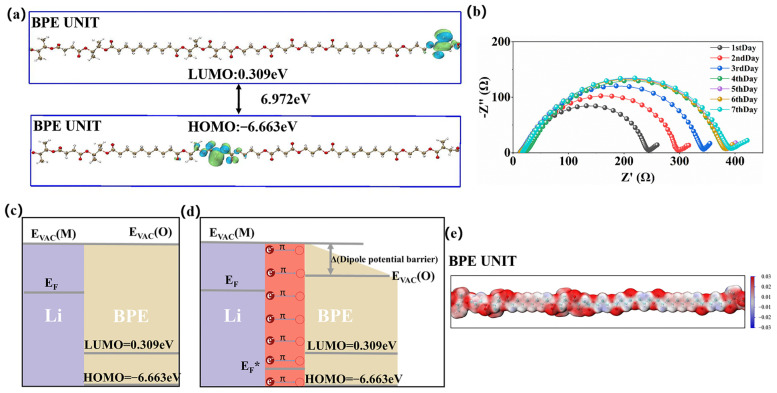
(**a**) HOMO and LUMO levels of BPE, (**b**) Li/BPSPE_44.4_/Li cell AC impedance spectrum at different number of days of standing, (**c**–**d**) energy relationship between BPE and Li metal: (**c**) without interface dipole and (**d**) with interface dipoles, (**e**) the electrostatic potential of BPE obtained by simulation calculation.

The frontier molecular orbitals, specifically the highest occupied molecular orbital (HOMO) and lowest unoccupied molecular orbital (LUMO), can provide valuable insights into the electronegativity of polymers [45]. To examine the electronegativity of the polymer electrolyte and explore the relationship between the interface energy and lithium metal, the HOMO–LUMO energy levels (Figure 6a) of BPE were determined through simulation calculations [46]. The interface between lithium metal and BPE was analyzed using energy band theory [47]. The LUMO and HOMO levels of BPE were 0.309 and −6.663 eV, respectively. The wide energy gap of 6.972 eV indicates that BPE is an insulator without the characteristic of electron conduction [48]. Figure 6c and d show the interface energy of BPE and lithium metal based on the Schottky–Mott model. According to the Schottky–Mott model theory, lithium and BPE share the same vacuum energy level. However, BPE, serving as an organic material that acts as a lithium-ion transport layer (shown by its high ion conductivity), is categorized as a polar molecule. On the lithium metal and BPE interface, a dipole is inevitably generated as shown in Figure 6d; the vacuum level of original interface damage is observed. The interface of the dipole potential barrier Δ and the dipole barrier is related to the work function of lithium metal, which is 2.9 eV, indicating the ease of providing electrons. According to the Koopmans theorem, the electronegativity of BPE is 3.177 eV [47], which is a strong electron-deficient compound. And the contact between the compound and the metal creates a large negative dipole. Additionally, as shown in Figure 6d, a charge transfer complex (highlighted in orange) is generated after the charge transfer between lithium and BPE in the ground state at the interface. When charge transfer complex reaches equilibrium, the Fermi energy level (E_F_^*^) on the Li/BPE interface remains stable and is lower than the LUMO level. Both the hole and electron barrier decrease, which is beneficial for interface stability. Figure 6b presents the AC impedance spectra of the Li/BPSPE_44.4_/Li electrochemical cell over different days of operation. The results illustrate that the impedance progressively increases during the initial four days, indicating an interfacial wetting process between BPE and lithium metal. After 4 days of operation, the impedance becomes relatively stable, indicating the gradual formation of a stable complex at the interface following the contact between lithium metal and BPE. This observation is consistent with the simulation results. The molecular electrostatic potential (MEP) is primarily employed to identify molecular interactions and predict the relative positions of nucleophilic and electrophilic attacks. The negative region of the MEP is associated with the attraction of lithium ions due to the density of electrons clustered in the molecule (pink shading) and is the preferred site for nucleophilicity. According to Figure 6e, it is evident that in the BPE unit, the negatively charged regions are located on the oxygen atoms distributed between the long carbon chains. This corresponds to the high ionic conductivity and high electrochemical stability of BPE [49].

**Figure 7:**
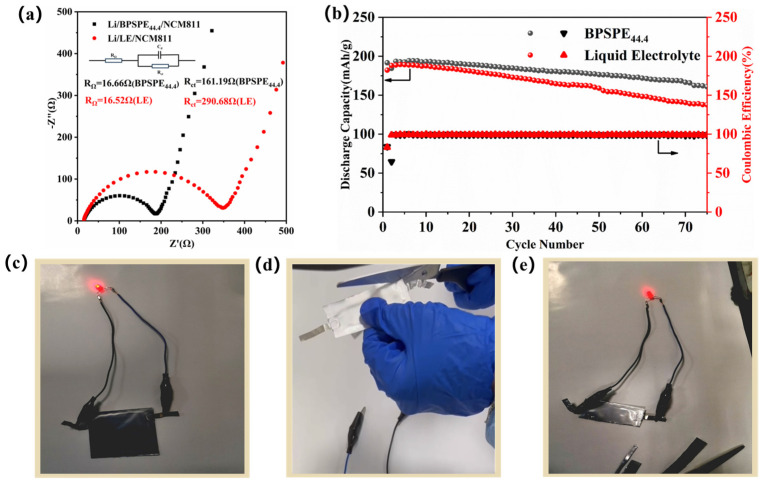
(**a**) EIS impedance spectra of Li/Liquid electrolyte (80 μL)/NCM811 cells and Li /BPSPE_44.4_/NCM811 cells before electrochemical cycling test (at room temperature), (**b**) long cycling performance of Li/Liquid electrolyte (80 μL)/NCM811 cells and Li/ BPSPE_44.4_/NCM811 cells at a 0.1 C in room temperature, (**c**–**e**) graphite/BPSPE_44.4_/LiCoO_2_ (4.5 V) all solid-state soft pack battery lighting small bulb experiment: (**c**) complete soft pack battery, (**d**) the shearing process of the software package, (**e**) defective soft-pack battery.

Figure 7a displays the electrochemical impedance spectrum of BPSPE_44.4_ and liquid cell (80 μL) at room temperature, and it can be seen that the Li/BPSPE_44.4_/NCM811 cell exhibits lower impedance values, indicating that BPSPE_44.4_ provides a lithium-ion transport channel comparable to that of liquid electrolytes. Figure 7b shows the long cycle diagram of the NCM811/BPSPE_44.4_/Li solid cell and the NCM811/Liquid Electrolyte (80 μL)/Li liquid cell at a 0.1 C current density at room temperature. Evidently, the cut-off voltage was 3–4.3 V, and the NCM811/Liquid Electrolyte (80 μL)/Li cell exhibited a discharge capacity of 182.2 mAh g^−1^. The NCM811/Liquid Electrolyte (80 μL)/Li cell exhibited a rapid decay rate during cycling. After 100 cycles, the discharge capacity declined to 57.9 mAh g^−1^, attributed to the poor interface contacts between Li and the limited amount of electrolyte. The battery equipped with BPSPE_44.4_ exhibited an initial discharge capacity of 191.7 mAh g^−1^. After 100 cycles, the capacity of the NCM811/BPSPE_44.4_/Li solid battery exceeded 128.2 mAh g^−1^, with a Coulomb efficiency over 98%. As shown in Figure S2, under the same conditions, BPSPE_44.4_ batteries exhibited higher first charge capacity than liquid batteries. This indicates that BPSPE_44.4_ has good contact with the electrode interface as well as good cycling stability.

To further demonstrate the practicality and safety of BPE all-solid-state battery, Graphite/BPSPE_44.4_/LiCoO_2_ all-solid-state flexible package battery was prepared [50,51]. As shown in Figure 7c–e, the red light-emitting diode (LED) lamp is successfully lighted by the solid-state pouch cell, even after shearing at room temperature (the battery clipping process is detailed in Video S1 of Supplementary Materials). This demonstrates the high safety of Graphite/BPSPE_44.4/_LiCoO_2_ solid-state batteries.

## 4. Conclusions

In this study, we successfully synthesize biobased high-ester-based-density amorphous state conducting lithium-ion substrates (BPEs) using a random copolymerization method. The impact of ionic concentration on ionic migration and ionic conductivity of amorphous molecules with high ester group density is investigated from the perspective of molecular structure. BPSPEs achieve high ionic conductivity (1.03 mS cm^−1^ 25 °C) and low ionic activation energy at lithium salt concentrations of up to 44.4 wt.%. Additionally, the long carbon chain structure enables BPSPE to demonstrate an excellent electrochemical window (5.08 V, vs. Li/Li^+^) and long-term cycle stability to lithium metal. NMR carbon spectroscopy shows that lithium ions preferentially form complexes with sterically unhindered ester groups. In addition, the stabilization of polymer and lithium metal at the interface is analyzed from the energy band point of view in conjunction with simulations. This investigation provides a novel perspective for the advancement of ester polymer electrolytes as a high-performance electrolyte for lithium batteries.

## Data Availability

The data that support the findings of this study are available from the corresponding author (X.P.) upon reasonable request.

## Supplementary Materials

The following supporting information can be downloaded at: https://www.mdpi.com/article/10.3390/nano13233027/s1, Table S1: Comparison of thermodynamic properties, activation energy, and ionic conductivity of electrolytes with different lithium concentrations; Figure S1: EIS curves of SS/BPSPE_44.4_/SS batteries at different temperatures; Table S2: Area comparison of different samples and different peak positions; Video S1: Graphite/BPSPE_44.4_/LiCoO_2_ (4.5 V) all solid-state soft pack battery shearing process; Figure S2: First charge–discharge curves for Li/BPSPE_44.4_/NCM811 cells and Li/Liquid electrolyte /NCM811 cells.
