# Supplementary material for: A Biodegradable Polyester-Based Polymer Electrolyte for Solid-State Lithium Batteries
Source: Nanomaterials (Basel). 2023 Nov 27;13(23):3027. doi: 10.3390/nano13233027 (PMC10707830; doi:10.3390/nano13233027)
Supplement: Supplementary file 1 [file nanomaterials-13-03027-s001.zip › nanomaterials-2692982 SI.pdf]

nanomaterials

MDPI

Supplementary Materials

A biodegradable polyester-based polymer electrolyte for solid state lithium batteries

Chenxia Tang [1], Zhiyu Xue [1], Shijie Weng [1], Wenjie Wang [1], Hongmei Shen [1], Yong Xiang [1,2,3], Le Liu [4] and Xiaoli Peng [1,2,3,] *

1 School of Materials and Energy, University of Electronic Science and Technology of China, Chengdu 611731, Sichuan, China.
2 Advanced Energy Research Institute, University of Electronic Science and Technology of China, Chengdu 611731, Sichuan, China.
3 Sichuan Provincial Engineering Research Center of Flexible Display Material Genome, University of Electronic Science and Technology of China, Chengdu 611731, Sichuan, China.
4 School of Mechanical and Electrical Engineering, Wuhan Institute of Technology, Wuhan 430200, Hubei, China.
* Correspondence: pxl@uestc.edu.cn

Table S1. Comparison of thermodynamic properties, activation energy, and ionic conductivity of electrolytes with different lithium concentrations

Sample types	T_g (°C) (via DSC)	Conductivity (×10^{-3} S cm^{-1})	E_a (KJ mol^{-1})	E_a (10^{-3}eV)
BPE	-60.2	——	——	——
$BPSPE_{16.7}$	-60.9	0.023	0.722	7.488
$BPSPE_{44.4}$	-61.2	1.030	0.496	5.150
$BPSPE_{58.3}$	-61.4	0.368	0.570	5.926
$BPSPE_{61.5}$	-61.5	0.391	0.697	7.243

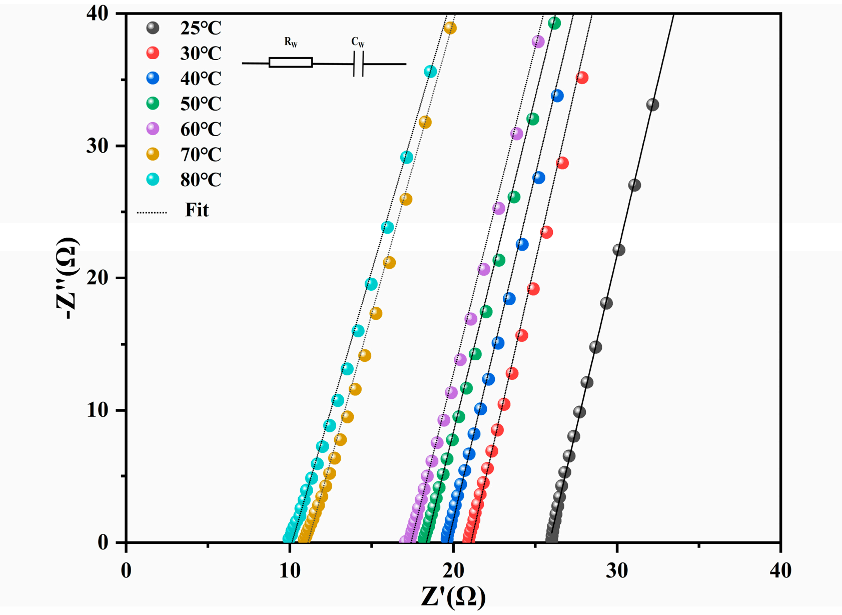

Figure S1. EIS curves of SS / $BPSPE_{44.4}$ / SS batteries at different temperatures

Table S2. Area comparison of different samples and different peak positions

Peak Position	Chemical Shift (ppm)	BPE (10^{-4})	$BPSPE_{44.4}$ (10^{-4})
1	24.8	1.51	0.43
2	25.3	2.67	0.96
3	27.9	1.40	0.57
4	29.1	6.87	3.09
5	29.6	1.44	4.65
6	30.8	0.42	6.82
7	34.3	1.49	0.79
8	60.7	0.49	0.44
9	61.4	1.67	0.54
10	63.8	0.50	0.62
11	64.3	1.57	0.70
12	96.2	0.53	0.68
13	172.3	1.50	0.63
14	173.9	1.14	0.32
15	175.7	-----	2.00
Total	-----	23.20	23.24

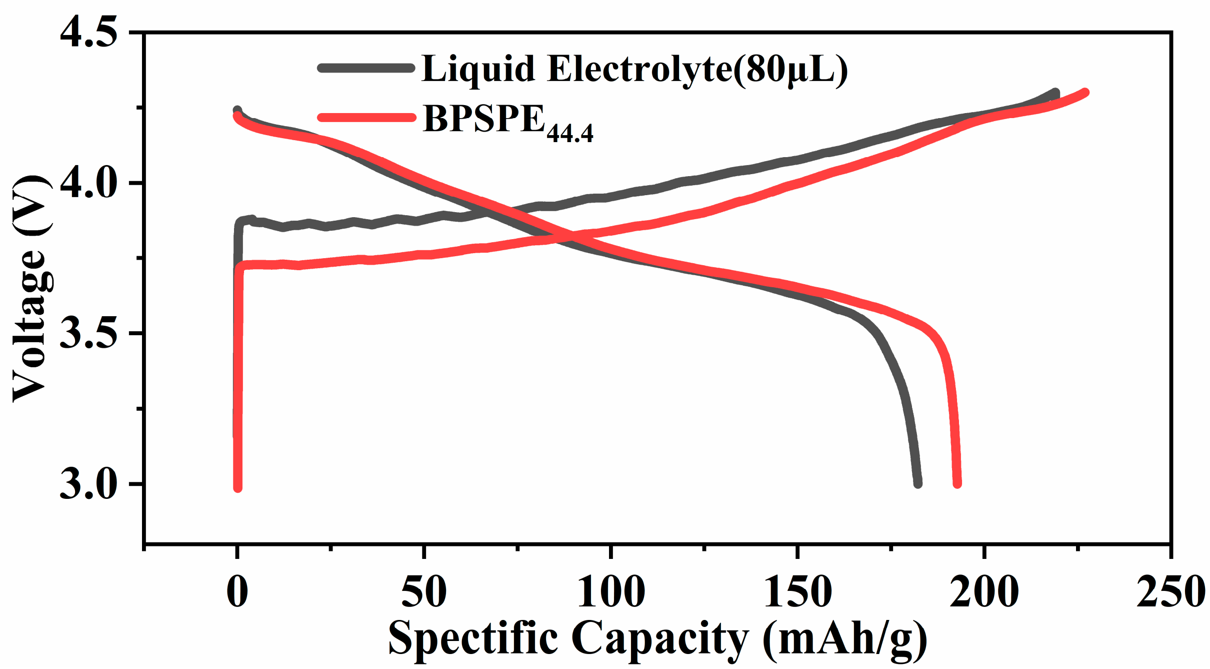

Figure S2. First charge-discharge curves for Li / $BPSPE_{44.4}$ / NCM811 cells and Li / Liquid electrolyte (80μL) / NCM811 cells.
